# An investigation of the relationship between cyberbullying, cybervictimization and depression symptoms: A cross sectional study among university students in Qatar

**DOI:** 10.1371/journal.pone.0260263

**Published:** 2021-12-09

**Authors:** Sara Mohamed Alrajeh, Huda Mohammed Hassan, Aisha Salim Al-Ahmed, Diana Alsayed Hassan

**Affiliations:** Department of Public Health, College of Health Sciences, QU Health, Qatar University, Doha, Qatar; University of Toronto, CANADA

## Abstract

**Background:**

Cyberbullying is a modern form of bullying that could be practiced electronically or on the internet. It is related to different mental health issues such as depression, which can affect both the cyberbully and the victim. Although a few studies have been conducted regarding the prevalence of cyberbullying and cyber-victimization among the younger generation in Qatar, no studies have been conducted among young adults despite studies showing that they are also prone to cyberbullying.

**Methods:**

This is a cross-sectional study to investigate the prevalence and the relationship between cyberbullying, cyber-victimization, and depression symptoms among Qatar University students. A self-administered close-ended electronic questionnaire was used to assess student’s cyberbullying/cyber-victimization behaviors and depression symptoms. The Revised Cyberbullying Inventory scale (RCBI-II) and Patient Health questionnaire-9 (PHQ-9) were utilized to measure involvement in cyberbullying and depression symptoms, respectively. A total of 836 students participated in the study. Pearson Chi-Square test and binary logistic regression were conducted to analyze the data.

**Results:**

Results indicated the majority of students have been involved in cyberbullying as follows: 6.8% cyberbullies, 29.2% cybervictims, 35.8% cyberbully-victims, and 28.2% not involved in either. Approximately 50% of the students scored a ten or higher on the PHQ9 test indicating symptoms of depression. Moreover, significant associations were found between cyberbullying experiences and gender (p = 0.03), depression and gender (p = 0.046), and between cyberbullying experiences and depression (p<0.001).

**Conclusion:**

Our findings indicate that among Qatar University students, cyberbullying and cyber-victimization are prevalent behaviors that could be associated with the high reported rates of depression symptoms.

## 1. Introduction

Over the past few years, cyberbullying has emerged as a social and health concern and has been recognized as a public health issue [1, 2]. Cyberbullying is a form of bullying carried out by an individual or a group of perpetrators through electronic or digital media with the intention to harm others [3, 4]. Another formal definition presented by the Cyberbullying Research Center is **“**willful and repeated harm inflicted through the use of computers, cell phones, and other electronic devices**”** [5]. Unlike traditional bullying, which occurs face-to-face, cyberbullying is not constricted by proximity to the bully, place, or time and the perpetrator can remain anonymous while engaging in the behavior creating a power imbalance between the bully and the victim. With the fast paced development in technology, internet penetrance, and the availability of different modern hand-held devices, cyberbullying has become an easily practiced behavior and a widely growing phenomenon worldwide [1, 3].

Contrary to the common belief, cyberbullying is not only an emerging issue among adolescents, but it is also a growing phenomenon among all age groups. A few studies examined the prevalence of cyberbullying and cybervictimization among various age groups and found that cyberbullying is reported at similar rates among adolescents and young adults [6, 7]. For example, Kim et al. (2018) reported that cyber-victimization rates among adolescents (15–17 years old) and young adults (18–25 years old) in their study were 12.2% and 10.4%, respectively. Furthermore, a study by Vranjes et al. (2018) found that 18% of adults experienced work-related cybervictimization compared to adolescents who reported a rate of 27%. However, because cyberbullying has been traditionally approached as an adolescent issue, it has not been sufficiently researched among adults. Furthermore, many of these studies explored cyberbullying of adults at the workplace [8–11]. However, there’s emerging research that has been focusing on studying cyberbullying among university students as young adults who remain vulnerable to cyberbullying consequences.

Moreover, there are studies indicating that adolescents who were involved in cyberbullying whether as a victims or perpetrators, continue to engage in this repeated pattern in early adulthood [12, 13]. Chapell et al. (2006) found that over 70% of undergraduate students in their study who reported being bullied in school, and over 50% who reported being bullies in school, reported bullying others while in university. A different study found that 50% of university students who reported being victims, also reported being victims while in high school [13]. These findings indicate that the vicious cycle of cyberbullying has a tendency to persist.

Studies that were carried out on university students in many parts of the world revealed a prevalence of cyberbullying ranging between 11% and as high as 55% [14–16]. While the topic of cyberbullying has been explored worldwide, fewer studies have been conducted in the Middle East region. Despite the 100% internet penetration in Qatar, the highest in the Middle East, and the existence of initiatives and legislation that criminalizes cyberbullying, there is a lack of current research and awareness on cyberbullying in Qatar [17]. This, however, is not indicative that the problem does not exist. The Global School-based Student Health Survey (GSHS) conducted in 2011 in Qatar revealed that over 43% of surveyed youth reported being bullied on at least one occasion in the previous month [18]. Although useful, this data does not provided a comprehensive depiction of the issue nor the questions asked in the survey accurately capture the repetitive nature of bullying [17]. Research data on cyberbullying in Qatar is very scarce as well. An online survey conducted by Microsoft (2012) among 8–17 years old in 25 countries found that Qatar had the 19th highest rate of cyberbullying out of 25 countries worldwide included in the study. Furthermore, bullying someone online among Qatari youth was reported to be higher than average (32% vs. 24%, respectively) while being bullied by someone else was lower than average (28% vs. 37%, respectively). The rate of students who reported being worried of online bullying was 24% while only 50% reported knowing what online bullying is [19].

Despite the fact that cyberbullying is relatively a new phenomenon, multiple studies demonstrated the negative effects of cyberbullying on the physical and psychological aspects of health, as well as the academic performance of students involved [20–22]. Studies that focused on the psychological effects of cyberbullying found that victims reported high rates of depression and other mental health issues such as anxiety, low self-esteem, anti-social behaviors, and in some cases even suicide [23–26]. However, victims of cyberbullying are not the only ones being affected. In fact, the harms of cyberbullying extend to perpetrators as well. Studies show that perpetrators reported high stress levels, lower grades, higher odds for depression and alcohol abuse [16, 27] Other studies that compared the outcomes of traditional bullying to cyberbullying demonstrated that the negative outcomes of cyberbullying can extend beyond those of traditional bullying. A study found that being involved in cyberbullying as a victim or perpetrator contributed to predicting depressive symptoms and suicide ideation more than in those exposed to traditional bullying [28]. Moreover, these negative outcomes can extend into adulthood up to four decades beyond the cyberbullying experience putting these individuals at risk for poorer health outcomes as adults [29].

Depression, a leading cause of disability, is one of the most common mental health disorders among university students and is on the rise. A study that examined data collected by the American College Health Association from 454,029 university students in the United States found that diagnosis and treatment of depression increased from 9% in 2009 to 12.2% in 2015 [30]. In a systematic review, depression prevalence among university students was found to be higher than that in the general population [31]. Multiple studies conducted worldwide revealed that the prevalence of depression among university students ranged between 15.6% and as high as 33% [32–35]. The prevalence of depression among Qatari adults is 13.5% [36], which is comparable to other developed countries in the region. However, the prevalence of depression among adolescents attending secondary school is alarmingly 34.5% [37], while depression among university students in Qatar was nearly three times higher than the students in the United States (32% vs. 12.8%, respectively) [38].

This cross- sectional study aims to fill the gaps in research by investigating the prevalence and relationship between depression symptoms and cyberbullying experiences among young adults in a university setting.

## 2. Materials and methods

### 2.1 Study design, sampling, and participants

This is a cross-sectional study to investigate the relationship between cyberbullying, cyber-victimization and depression symptoms among university students at Qatar University. Qatar University, established in 1977, is the national and primary higher education institution in Qatar. The university houses ten Colleges with a diverse student body of over 20,000 students from Qatar and other countries. In 2019–220, 66% of registered students were Qatari, 34% non-Qatari, 78% females, and 22% males. The majority of the non-Qatari students are Arabs from different nationalities and from the Indian subcontinent [39].

We adopted a universal sampling method and sent an electronic self- administered close-ended anonymous survey to all 20,000 students who were actively enrolled at Qatar University in Spring 2019. Students had the option to take the survey in English or Arabic. We received a total of 870 survey responses of which we excluded 34 due to incomplete answers resulting in our final sample of 836 students. The low response rate could be due to the fact that data was collected towards the end of the semester; a busy period for students. Qatar University Institutional Review Board approved the study.

### 2.2 Survey

The survey included three sections:

**Demographics:** including age (under 18, 18–24, 25–34, 34 and above), sex (male/female), and college level (5 levels), and nationality (Qatari, non-Qatari).

**The Revised Cyber Bullying Inventory–II (RCBI-II) Questionnaire**, which measures cyberbullying and cyber-victimization (40). The RCBI-II tool is comprised of 10 items that participants had to answer twice. Once for if they had participated in the listed cyberbullying behavior in the “I did this-bully scale” section and a second time for reporting if the behavior was performed against them as a cybervictim in the “It happened to me- victim scale” section. The RCBI-II was scored on a 4-piont scale ranging from (1) “never,” (2) “once,” (3) “twice or three times,” (4) “more than three times”. The minimum possible score is 10 and the maximum possible score is 40. Based on students’ scores on this scale, they were then categorized into 4 categories of involvement: 1. cyberbullies: those who scored > 10 points in the cyberbullying section and a10 in cyber-victimization section, 2. cyber-victims: those who scored >10 in the cyber-victimization section and a 10 in cyberbullying section, 3. cyberbully-victims: those who scored >10 points in both cyberbullying and cyber-victimization section, and 4. Not involved: those who scored 10 points in both cyberbullying and cyber-victimization section [40]. The RCBI-II was translated using the forward-backward translation method to ensure the questionnaire is culturally appropriate and was piloted with a group of 10 university students. In our study, the calculated Cronbach’s alpha coefficient for the cyberbullying and the cyber-victimization scale were .8 and .85, respectively.

**The Patient Health Questionnaire-9 (PHQ-9)** was used to measure depression symptoms (Kroenke et al., 2001). It is comprised of 9 items and scored on a Likert scale ranging from “not at all = a score of 0” to “nearly every day = a score of 3”. The total scores can range from 0 to 27 with higher scores indicating a higher depression severity [41].

We used the PHQ-9 to measure symptoms and severity of major depressive disorder. Based on students’ total scores, they were categorized into six groups representing the severity of symptoms: none (0), minimal (1–4), mild (5–9), moderate (10–14), moderately severe (15–19), and severe (20–27). However, for the analysis part of our study, participants were further classified into two groups making depression a binary variable (depressed, not depressed). Out of a total of 27 possible points, a cutoff of 10 points or higher was used to classify participants with at least moderate depression and those who scored under 10 points were classified as the group with no depression [16, 42]. PH-Q9 has been validated in previous studies among university students [43, 44]. An Arabic version has also been validated among university students in Saudi Arabia [45]. It is important to note that PHQ-9 tool was not used for diagnosis purposes in our study.

### 2.3 Statistical analysis

For the categorical variables (cyberbullying experience and depression), descriptive analyses included calculations of means, standard deviations (SD), medians and percentages. Statistical comparisons of groups were then conducted using Pearson Chi-Square test for categorical data. Binary logistic regression analysis to explore the relationship between depression and a set of independent variables (cyberbullying experiences, age, gender, college and college level) was used. The alpha level was set at five percent for statistical significance while performing two-sided hypotheses testing. IBM SPSS statistics version 25 (Armonk, NY: IBM Corp.) was used for analyses.

## 3. Results

Demographics and descriptive statistics are shown in Table 1. The sample size of the study was 836 students. Results showed that the majority of student respondents were females (81.5%) between the ages of 18 to 24 years old (75.6%). When examining student categories based on their cyberbullying experiences, the majority reported experiencing both being a bully-victim (35.8%), followed by victims only (29.8%), then by not involved (28.2%). Only 6.8% of students reported being a cyberbully. As for our dependent variable (depression), based on a cutoff total score of 10 or higher, students were categorized into 2 groups (418 depressed and 418 not depressed). More than half of female students (51%) scored at or above the cutoff score compared to 45.8% of male students, meeting the criteria for depression. When examining the severity of depression symptoms, we found that the majority of students (96.3%) reported symptoms of depression as follows: minimal (13.6%), mild (32.7%), moderate (24.5%), and severe (10.6%).

**Table 1:** Demographics and descriptive statistics.

	N (%)
Age	
Under 18	13 (1.6)
18 to 24	632 (75.6)
25–34	149 (17.8)
Over 34	42 (5.0)
Gender	
Male	155 (18.5)
Female	681 (81.5)
Nationality	
Qatari	457 (54.7)
Non-Qatari	379 (45.3)
College Level	
First year	226 (27.0)
Sophomore	192 (23.0)
Junior	183 (21.9)
Senior	172 (20.6)
Graduate level	63 (7.5)
Cyberbullying experiences	
Cyberbullies	57 (6.8)
Cybervictims	244 (29.2)
Cyberbully-victims	299 (35.8)
Not Involved	236 (28.2)
Depression symptoms	
None	31 (3.7)
Minimal	114 (13.6)
Mild	273 (32.7)
Moderate	205 (24.5)
Moderately severe	124 (14.8)
Severe	89 (10.6)
Depression	
Scored ≥10 on PHQ-9	418 (50)

To determine the interrelationship between cyberbullying, cybervictimization and depression, Pearson’s correlation analysis was performed and revealed significant positive associations, although not strong, between cyberbullying, cybervictimization, and depression symptoms as shown in Table 2.

**Table 2:** Interrelationship between cyberbullying, cybervictimization, and depression.

	1	2	3
1. Cyberbullying		.503**	.205**
2. Cybervictimization			.385**
Depression symptoms			
Mean	11.56	13.75	10.63
Standard Deviation	3.11	4.86	6.43

** Correlation significant at the 0.01 level (2-tailed).

**Fig 1:**
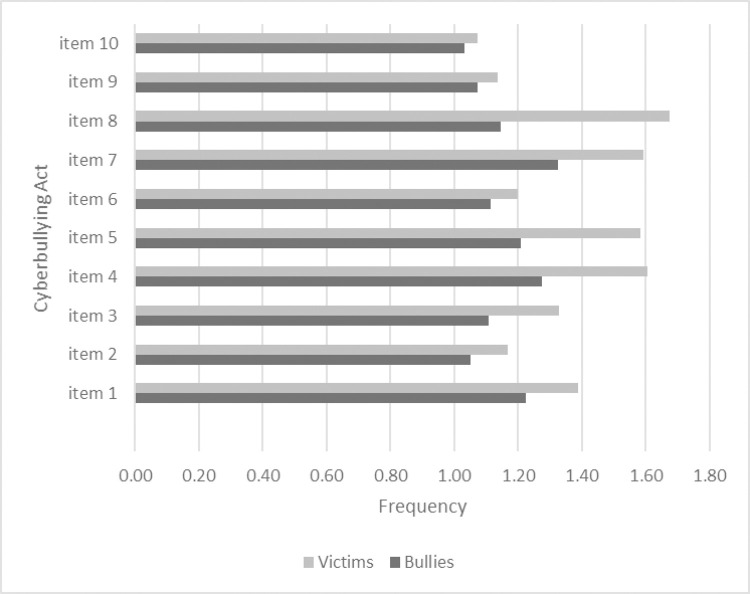
1. taking over the password of someone’s account, 2.using someone’s account without his/her permission and publishing humiliating posts, 3.threatening someone, 4.insulting someone, 5. sending embarrassing and hurtful messages, 6.sharing an inappropriate photo or a video of someone without his/her permission, 7.sharing a secret with others without the permission of the owner, 8.spreading rumors, 9.creating an account on behalf of someone without letting him/her know and acting like the account’s owner, 10.creating a humiliating website.

When examining the reported frequency of these online behaviors, we observed that among victims of cyberbullying, the most reported behaviors were spreading rumors, insulting someone, and sharing a secret with others without the permission of the owner. While among bullies, the most reported behaviors were sharing a secret with others without the permission of the owner, insulting someone, and taking over the password of someone’s account as seen in Fig 1

When examining gender differences, we found that among female students, 33.5% (228) fell in the cyberbully-victims category while only 6.8% [46] fell in the cyberbully category. Similar results were found among male students where the majority fell in the cyberbully-victims group (45.8%). Moreover, a Pearson Chi-square test revealed a significant association between cyberbullying and gender (p < 0.05). Being a cyberbully and a cyberbully-victim were more frequently reported among males while the proportion of women reporting being a victim was higher than men as seen in Table 3.

**Table 3:** Prevalence of cyberbullying experiences by gender.

	Cyberbullying experiences					
	Total	Bully	Victims	Bully-victim	Not involved	p
		N = 57	N = 244	N = 299	N = 236	
	N = 836	(6.8%)	(29.2%)	(35.7%)	(28.2%)	
	n %	n (%)	n %	n %	n %	
Gender						0.030
Male	155 100	11 (7.1)	36 (23.2)	71 (45.8)	37 (23.9)	
Female	681 100	46 (6.8)	208 (30.5)	228 (33.5)	199 (29.2)	

A Pearson Chi-square test was used to assess the significant association between gender and depression, and no significant association was found (p = 0.247) as seen in Table 4.

**Table 4:** Prevalence of depressive symptoms by gender.

Depressive Symptoms				
	Total	Depressed	Not depressed	P value
	N = 836	N = 418	N = 418	
	n %	n %	n %	
Gender	0.247			
Male	155 100	71 45.8	84 54.2	
Female	681 100	347 51.0	334 49.0	

The Patient Health Questionnaire-9 (PHQ-9), Pearson Chi-square test. Statistical significance p < 0.05.

A Chi-square test revealed a positive significant association between cyberbullying experiences and depressive symptoms (p<0.001) while the other variables (age, college, and college levels) had no significant relationship with depressive symptoms. However, binary logistic regression analysis demonstrated that the variable cyberbullying experience was significantly associated with depressive symptoms (p<0.001). The odds for depressive symptoms for students who were cybervictims and cyberbully-victims, were more than two times (aOR 2.497 [95% CI 1.704–3.659]) and three times higher (aOR 3.260 [95% CI 2.249–4.725]), respectively, when compared to students who were not involved, as shown in Table 5. It is important to note that among those not involved, 23.3% were depressed. Moreover, unlike in chi-square analysis, there was a positive significant association between gender and depressive symptoms (p = 0.046) as female students had an odds for depressive symptoms that is 1.489 higher than male students (aOR 1.489 [95% CI 1.007–2.228]) as seen in Table 5.

**Table 5:** Comparison of cyberbullying experiences, age, gender, college, college level and depressive symptoms.

	Depressive Symptoms			
	aOR	95% CI for EXP (B)		P
		Lower	Upper	
Cyberbullying experiences				< 0.001
Cyberbullies	1.709	0.933	3.131	0.083
Cyber-victims	2.497	1.704	3.659	< 0.001
Cyberbullies/cyber-victims	3.260	2.249	4.725	< 0.001
Age				0.085
Gender (male/female)	1.489	1.007	2.228	0.046
College				0.180
College level				0.417

## 4. Discussion

In our study, we document the prevalence of cyberbullying and shed light on one of the most prevalent mental health issues among university students; depression, which we found to be alarmingly high. We found that half of our student sample reported being depressed. When further looking into the severity of depressive symptoms, we found that almost all of our students reported some symptoms of depression ranging from minimal to sever.

Our study revealed that cyberbullying is a phenomenon more prevalent than expected, yet understudied in the region. We found that the majority in our sample identified as cyberbully-victims followed by cybervictims, while only a very small percentage identified as cyberbullies only. Furthermore, we found that cyberbullying is associated with depression, specifically in cybervictims and cyberbully-victims who have higher odds of depression than those not involved. Gender differences were also prominent in our study with females being more likely to be victims while males were more likely to be cyberbullies and cyberbully-victims.

### 4.1 Cyberbullying

Reported rates of cyberbullying experiences from our study are comparable to previous studies conducted among university students. Our study revealed that only 6.8% of surveyed students reported being cyberbullies as compared to the majority of students who identified as cybervictims or cyberbully-victims, as similarly found in other studies [13, 46–48]. The relative low percentage of students identifying as cyberbullies could be attributed to the fact that cyberbullying is a relatively new and underexplored topic in the local context and there is a potential lack of knowledge and understanding of cyberbullying among university students. This may result in misinterpreting behaviors related to cyberbullying; thus distorting ones self-perceptions related to being a bully. A study by Walker (2011), argues that it is possible that students have distorted perceptions related to online communication behaviors. These behaviors may not be acceptable in traditional face-to-face communication, but students might view them as an acceptable part of online social life. These perceptions could affect both perpetrators and victims [14]. Perpetrators may not be realizing the harm they are inflicting on others thinking that it is an acceptable behavior while victims may be underreporting cyberbullying incidences and not seeking the appropriate help they need. Francisco et al (2015), revealed that students tend to underrate their cyberbullying involvement as only 8% of their surveyed students perceived themselves as cyberbullies, 63.5% of students who were cyberbullied reported the bullying incident to a person of authority, and only 59.1% reported asking someone trustworthy for help [48].

While the interaction of various factors that contribute to aggressive behavior online has been explained through theories such as the General Aggression Model [49, 50], there’s a lack of theories that specifically address cyberbullying. To our knowledge, Barlett and Gentile Cyberbullying Model (BGCM) is the only theoretical model that explicitly explains the mechanisms of cyberbullying [51]. The BGCM posits that perceptions of anonymity in online interactions facilitate aggressive online behaviors [52]. The BGCM also explains that contrary to traditional bullying, physical muscularity is an irrelevant factor in individuals who engage in cyberbullying perpetration [53]. The model suggests that once individuals form accepting perceptions towards anonymity and irrelevance of muscularity in online environments, positive attitudes towards cyberbullying are formed, which subsequently predict cyberbullying perpetration [54]. While our study focused on the relationship between cyberbullying experiences and depressive symptoms, the BGCM helps shed some light on the possible factors contributing to engaging in cyberbullying behaviors that warrant further examination in future studies with a consideration of the different cultural context. Furthermore, numerous studies have identified risks and protective factors against cyberbullying and cybervictimization. A meta-analysis of the predictors of cyberbullying perpetration and victimization [55] identified that offline bullies (*r* = .39) and externalizing problems (*r* = .31) as the strongest predictors to cyberbullying. The study also found that being an offline victim to traditional bullying was the strongest predictor of cyberbullying victimization (*r* = .42). These results indicate that the cycle of bullying transitions and continues from a traditional bullying setting to cyberbullying which are emphasized by other studies as well [56, 57]. Another systematic review of meta-analyses identified positive communication, positive school climate, and school safety as protective factors against victimization while low frequency of technology use was identified as a protective factor against being a cyberbully [58]. While our study did not examine risk and protective factors, these findings could guide designing prevention programs.

### 4.2 Gender differences

In our study, we found that there is a significant association between gender and cyberbullying experiences (p = 0.030) as males were more likely to be cyberbullies and cyberbully-victims while females were more likely to be cybervictims. Gender differences in cyberbullying among adolescents and young adults have been explored in multiple studies, however, these studies revealed mixed findings. There are a few studies with similar result to ours reporting that females are more likely to be victims of cyberbullying and that males are more likely to engage in cyberbullying behavior [13, 59–61]. Contrary to our study, other studies found that cybervictimization is higher among males [15] while other studies found no gender differences [5, 62–64]. As there is no clear consensus on gender difference in relation to cyberbullying experience, future studies in this area are necessary to understand the role gender plays in cyberbullying. Furthermore, most of the studies that we compared our findings to were conducted in western cultures as we only found one study that examined cyberbullying among university students in a country with a similar cultural context; the UAE [65]. Nevertheless, that study only looked into students’ perceptions related to cyberbullying. An important element in future studies should be to investigate how gender roles within the context of culture influence cyberbullying behavior.

### 4.3 Depressive symptoms

In our study, the majority of students scored above the minimal depressive symptoms threshold. A similar finding was reported in a study that compared the rates of depression between university students in Qatar, Lebanon, and the United States. The study found that depression rates among the students in Qatar was nearly three times higher than the students in the United States (32% vs. 12.8%, respectively) [38]. Furthermore, in our study, a significant association was found between cyberbullying experiences and depression, which is consistent with what other studies found. Additionally, we found that being a cybervictim and cyberbully-victim had a significant association with depression compared to those not involved. This finding has been reported in other studies that also found significantly higher depression rates among adolescent cybervictims and cyberbully-victims. A longitudinal study by [47] that measured depression among adolescents at baseline and at a one-year follow-up found that at follow-up, both cybervictims and cyberbully-victims were twice as likely to report depressive symptoms compared to those uninvolved. Similar to our finding, their study found no significant difference in depressive symptoms among the cyberbully group. However, different results were reported in another study among female university students [16]. The study reported that when compared to uninvolved students, cyberbullies and cyberbully-victims had more than four times and three times higher odds for depression respectively, while victims had no significant association with depression. These findings, although contradicting, strongly support the hypothesis that a relationship exists between cyberbullying and depression. It is important to note that examining the relationship between depression and cyberbullying through a bidirectional lens has gained an interest in research. Multiple studies found that the relationship between cyberbullying and depression among adolescents can be reciprocal [66–68]. In one of the studies, the authors found that cyberbvictimization can lead to depressive symptoms and, in turn, depressive symptoms increase the chances of being a cybervictim [66]. The authors further explained that depressed adolescents may exhibit preferences to isolation and may lack social skills that could make them more vulnerable to being a victim of cyberbullying.

### 4.4 Limitations

One of the main limitations in this study is that we relied on a cross-sectional study design, which prevents us from identifying a causal relationship between our variables, therefore, longitudinal studies should be carried out in the future. Another limitation is that the data was collected using a self-reporting online questionnaire, which might result in lack of credibility in the answers due to social desirability and recall bias. This may have resulted in underestimating or overestimating the variables measured. Thus, future studies should employ diverse assessment tools and methodologies. Additionally, because cyberbullying, a relatively new and less explored topic, and depression, are both considered taboo topics in the local context, need to be further explored through the lens of culture, which can possibly be accomplished by carrying out longitudinal and qualitative studies that also take into account previous bullying experiences.

## 5. Conclusion and future research recommendations

This study sheds light on an important phenomena facing young adults as it offers valuable insights and information to professionals in higher education. We suggest that future efforts focus on increasing awareness of cyberbullying and its real consequences. In addition to continuing to explore the pathways between traditional bullying and cyberbullying and the factors contributing to this problem, based on evidence from the field, we recommend approaching this issue holistically through continuous efforts. We also recommend that any activities or initiatives designed to prevent cyberbullying must be built on sound theoretical frameworks to insure the success and sustainability of these efforts. We recommend that efforts should focus on increasing awareness across the educational lifespan, as more and more individuals from all age groups continue to be exposed and affected by cyberbullying due to the narrowing of the “digital age divide” idea [69]. Preventive efforts should be initiated in schools at an early age and imbedded at the university level as well. These efforts should include not only administrators and students, but also families who are still a vital element in the lives of university students and were found to be a protective factor against cyberbullying [70, 71].

As more and more research is highlighting the continuous cycle of bullying, we recommend that programs in schools incorporate social-emotional health and emotional intelligence principles as victims learn to disguise and internalize their feelings at a young age [71]. Programs should focus on teaching students 1) digital citizenship [72] 2) communication skills and 3) exercising empathy. Equally as important is to focus on students at risk for victimization and build their self-efficacy by providing them with 4) coping skills needed in such situations. Incorporating these four components in prevention programs, in addition to parental involvement, were found to significantly reduce cyberbullying and cybervictimization in youth [73].

While a few studies and reviews have examined the effectiveness of cyberbullying prevention programs in school age children, literature evaluating prevention interventions at the university level is scarce. Additionally, most of these studies have been conducted in the west while local and regional studies remain very scant. While policies and guidelines on cyber safety exist in Qatar, the extent of their implementation and effectiveness have not been examined [17]. Hence, further studies are warranted to examine cyberbullying with a holistic lens to pave the path for developing socio-culturally compatible guidelines and interventions.

## Supporting information

S1 Data (SAV)
